# Risk factors for postoperative thrombosis-related complications in patients undergoing malignant brain tumor resection: a retrospective cohort study

**DOI:** 10.3389/fneur.2023.1108596

**Published:** 2023-04-18

**Authors:** Xiaoyuan Liu, Xingyue Zhang, Tingting Ma, Muhan Li, Liyong Zhang, Shu Li, Min Zeng, Ira S. Kass, Yuming Peng

**Affiliations:** ^1^Department of Anesthesiology, Beijing Tiantan Hospital, Capital Medical University, Beijing, China; ^2^Anesthesiology and Physiology and Pharmacology Departments, State University of New York Downstate Health Sciences University, New York, NY, United States

**Keywords:** malignant brain tumors, thrombosis, cerebral ischemia, deep vein thrombosis (DVT), fresh frozen plasma (FFP)

## Abstract

**Introduction:**

Patients with malignant brain tumors frequently exhibit hypercoagulation and are at a high risk of postoperative thrombosis-related complications. However, the risk factors for postoperative thrombosis-related complications remain unclear.

**Methods:**

In this retrospective, observational study, we consecutively enrolled elective patients undergoing resection of malignant brain tumors from 26 November 2018 to 30 September 2021. The primary objective of the study was to identify risk factors for a composite of three major adverse events including postoperative lower limb deep venous thrombosis, pulmonary embolism, and cerebral ischemia.

**Results:**

A total of 456 patients were enrolled in this study, where 112 (24.6%) patients had postoperative thrombosis-related complications, 84 (18.4%) with lower limb deep venous thrombosis, 0 (0.0%) with pulmonary embolism, and 42 (9.2%) with cerebral ischemia. In a multivariate model, age more than 60 years (OR: 3.98, 95% CI: 2.30–6.88, *P* < 0.001), preoperative abnormal APTT (OR: 2.81, 95% CI: 1.06–7.42, *P* = 0.037), operation duration longer than 5 h (OR: 2.36, 95% CI: 1.34–4.16, *P* = 0.003), and admission to ICU (OR: 2.49, 95% CI: 1.21–5.12, *P* = 0.013) were independent risk factors of the postoperative deep vein thrombosis. Intraoperative plasma transfusion (OR: 6.85, 95% CI: 2.73–17.18, *P* < 0.001) was associated with significantly increased odds of deep vein thrombosis.

**Conclusion:**

Patients with craniocerebral malignant tumors have a high incidence of postoperative thrombosis-related complications. There is an increase in the odds of postoperative lower limb deep venous thrombosis in patients; over 60 years old, with preoperative abnormal APTT, undergoing surgeries longer than 5-h, admission to ICU, or receiving intraoperative plasma infusion. Fresh frozen plasma infusion should be used more cautiously, especially in patients with a high risk of thrombosis.

## Introduction

Thrombosis, a severe postoperative complication, is a major concern following surgery. Patients with brain tumors, particularly those with gliomas or malignant tumors, are often considered to be at a high risk of thromboembolic complications (1, 2). The incidence of postoperative deep vein thrombosis was reported to be 10.3–21.3% in patients undergoing brain tumor resections (3, 4). In patients with malignant gliomas, the incidence increased from 16.1 to 33.3% (5, 6). Retrospective studies have reported a 0.5–1.5% incidence of pulmonary embolism after brain tumor resections (7, 8). Perioperative cerebral ischemia in patients with gliomas was reported as high as 27.7–50% (9, 10). The risk factors for postoperative venous thromboembolism and cerebral ischemia following brain tumor surgery included increased age (5, 7, 11, 12), prolonged operation time (5, 7, 11), abnormal preoperative coagulation function (5, 7), tumor size and location (10, 13), and intraoperative blood loss (10, 14).

Fresh frozen plasma is required to improve hemostasis when facing coagulation factors deficiency induced by excessive bleeding. However, fresh frozen plasma may be aggressively administered in some circumstances to reduce the massive bleeding in the operation field, although the volume of blood loss maybe even <20% of total blood volume, which predominantly occurred during malignant brain tumor resection. The balance between the risks and benefits of aggressive fresh frozen plasma administration during neurosurgery is not clear. One study reported that intraoperative fresh frozen plasma infusion increased the risk of postoperative deep vein thrombosis in patients undergoing extramedullary spinal masses resections (15), and the median estimated blood loss was higher in patients who received fresh frozen plasma transfusion. However, the impact of the aggressive use of fresh frozen plasma on postoperative thrombosis-related complications in malignant brain tumors remains unclear.

Therefore, we conducted this retrospective, observational study to explore the risk factors for thrombosis-related complications in patients who underwent malignant brain tumor resections.

## Materials and methods

### Study design and participants

This retrospective, observational cohort study involved patients who underwent elective intracranial malignant tumor resection from 26 November 2018 to 1 September 2021, at Beijing Tiantan Hospital, Capital Medical University. The study protocol was approved by the Medical Ethics Committee of Beijing Tiantan Hospital, Capital Medical University (KY2021-173-02, date of approval: 16 December 2021). Given the retrospective nature, the ethics committee waived the need for written informed consent. The article adhered to the applicable Strengthening the Reporting of Observational Studies in Epidemiology (STROBE) standards for observational studies (16). Inclusion criteria were adult patients (≥18 years) and elective resection of intracranial malignant tumor, including World Health Organization (WHO) grade III-IV glioma, metastatic carcinoma, lymphoma, and atypical meningioma. Exclusion criteria included recurrent tumors, preoperative radiotherapy or chemotherapy, and postoperative coagulation measurement unavailable.

### Data collection

Data were obtained from the medical records. The variables included age, gender, body mass index, blood type (ABO and Rh), preoperative comorbidities (diabetes, hypertension, hyperlipidemia, myocardial infarction, and cerebral infarction), leg paresis, preoperative Karnofsky Performance Status (KPS), intraoperative fluid volume (total fluid volume, crystal fluid, colloid fluid, urine volume, estimated blood loss, red blood cells, fresh frozen plasma, and platelet), duration of surgery, surgical position, perioperative coagulation-related medication (human fibrinogen, hemocoagulase, etc.), postoperative complications (pneumonia, hepatic dysfunction, renal dysfunction, central nervous system infection, anemia, and electrolyte disorder), pathological types of tumor, tumor WHO classification, admission to intensive care unit (ICU), duration of ICU stay, duration of hospital stay, and medical cost.

We also collected information about coagulation factors preoperatively and on postoperative day 1; these included fibrinogen degradation product (FDP), D-dimer, prothrombin time (PT), international standardized ratio (INR), activated partial thromboplastin time (APTT), and fibrinogen. We defined abnormal coagulation function as a coagulation index beyond the clinically recommended normal reference range.

### Anesthesia procedure

Routine monitoring included non-invasive blood pressure, electrocardiography, pulse oxygen saturation, end-tidal carbon dioxide partial pressure, and body temperature. Anesthesia was induced with sufentanil, rocuronium or cis-atracurium, and propofol or etomidate and maintained with propofol, sevoflurane, and remifentanil. Analgesia was supplemented with sufentanil to attenuate the potential pain stimuli, during skull pin fixation, scalp incision, and dura suture.

After tracheal intubation, the tidal volume was fixed at 6–8 ml/kg, and the respiratory rate was adjusted to maintain end-tidal carbon dioxide partial pressure at 30–35 cm H_2_O. The standard for blood transfusion protocol at our institution is based on the NICE guidelines (17). Aggressive fresh frozen plasma infusion is defined as fresh frozen plasma transfusion at an estimated blood loss of <20% of total blood volume. Blood volume was calculated using the Gilcher quintile method, which estimated that a male patient has a blood volume of 70 ml/kg, while a female patient has a blood volume of 65 ml/kg (17, 18).

### Outcomes

The primary outcome included those as follows: (1) postoperative deep vein thrombosis, assessed by a lower extremity venous ultrasonography manifesting an intravascular hypoechoic mass or a moderate echoic mass; (2) postoperative cerebral ischemia, located far from the tumor-surgery site, assessed by magnetic resonance imaging examination manifesting a hypertensive signal on the diffusion-weighted image or a restricted diffusion area on the apparent diffusion coefficient maps; (19) and (3) pulmonary embolism defined as a filling defect in the pulmonary artery found by computed tomography pulmonary angiogram. The study did not include diffusion restrictions related to the operative field and the edema zone in postoperative cerebral ischemia. The outcomes were collected before discharge.

The secondary outcomes included postoperative non-thrombosis complications (myocardial infarction and arrhythmias, pneumonia, abnormal liver and renal functions, central nervous system infection, anemia, and electrolyte disturbances, as defined in Supplementary Table 1), duration of stay in hospital and ICU, and hospitalization cost.

### Statistical analysis

The continuous normal and skewed distribution variables were reported as mean (standard deviation, SD) and median (interquartile range, IQR), respectively. The categorical variables were expressed as count (percent). Patients were divided into two groups according to whether they exhibited postoperative deep vein thrombosis or cerebral ischemia. The difference in normally distributed variables and outcomes between groups was compared using the independent sample *t*-test, while skewed variables were compared using the Mann–Whitney *U*-test. The chi-square or Fisher exact test was used for categorical variables.

Multivariable logistic regression models were used to assess the association between intraoperative infusion of fresh frozen plasma and thrombosis-related complications. Confounders were defined as the statistically (*P* < 0.10 in the univariable analysis) and clinically significant variables. The Hosmer–Lemeshow goodness-of-fit test and the area under the curve were used to assess the model fit. The variable inflation factor was used to assess the correlation between predictors. The results were expressed as odds ratio (OR) and 95% confidence interval (CI). For all outcomes, a *p*-value of ≤0.05 was considered to be statistically significant. Statistical software package SPSS Statistics (version 23.0) was used for analysis.

## Results

**Figure 1:**
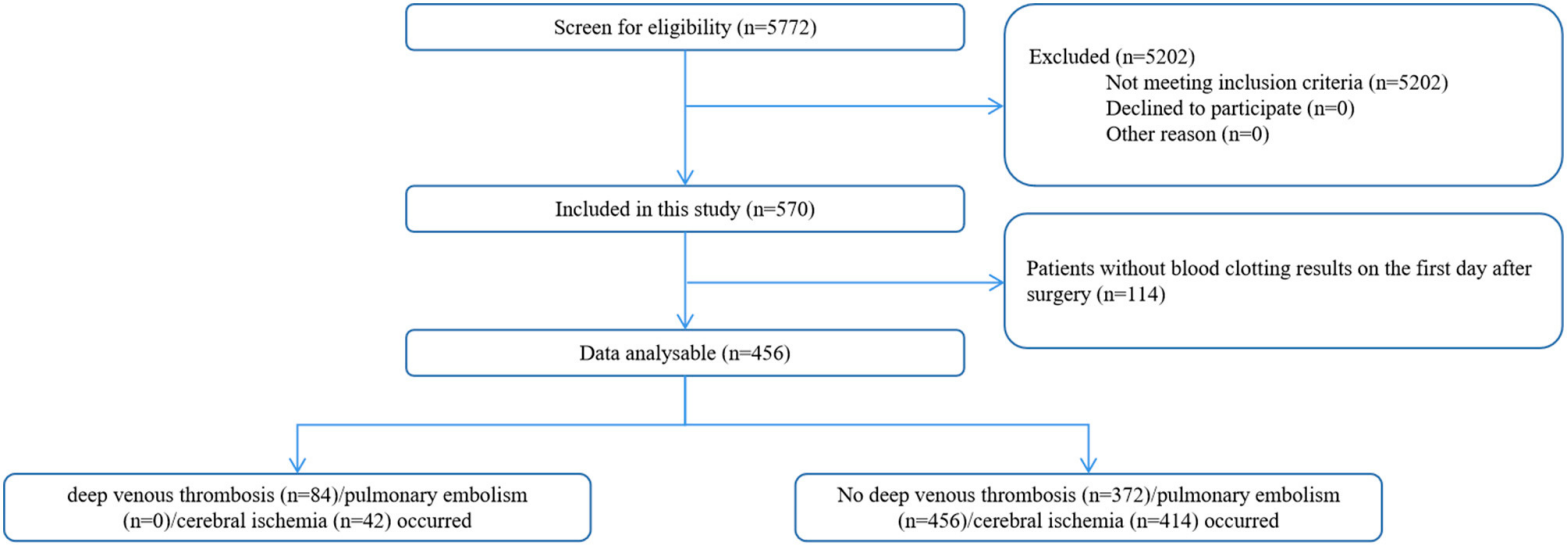
Flow chart.

Between 26 November 2018 and 1 January 2021, a total of 456 patients with elective intracranial malignant tumor resections were included in this study (Figure 1). Overall, thrombosis-related complications occurred in 112 out of 456 (24.6%) patients. Deep vein thrombosis was present in 84 out of 456 (18.4%) patients, and 42 out of 456 patients (9.2%) had cerebral ischemia. No patients had definite pulmonary embolism on postoperative computed tomography pulmonary angiogram. The characteristics of the postoperative thrombosis-related complications are presented in Table 1.

**Table 1:** Characteristics of the postoperative thrombosis-related complications.

	**Number of cases**	**Occurrence time (days after surgery), median (inter-quartile range)**
**Lower limb venous thrombosis**	84	5 (2–8)
**Lesions site**		
Muscular vein	82	–
Fibular vein	21	–
Posterior tibial vein	9	–
Popliteal vein	7	–
Femoral vein	1	–
Superficial femoral vein	1	–
**One or both lower limbs**		
One side	28	–
Both sides	56	–
**Treatment measures**		
Heparin	19	–
Tirofiban	10	–
No treatment	60	–
**Cerebral ischemia**	42	6 (3–7)
**Treatment measures**		
Urokinase, nimodipine, or edaravone	5	–
No treatment	37	–

The use of fresh frozen plasma was significantly higher in patients with postoperative cerebral ischemia (16.7 vs. 4.8%, *P* = 0.02) while urine volume was lower (4.14 vs. 5.04 mL/kg/h, *P* = 0.018) (see Supplementary Table 2).

For patients with postoperative deep vein thrombosis, the incidence of postoperative pneumonia, anemia, and electrolyte disturbances significantly increased (*P* < 0.05), as well as the proportion of ICU admission, the duration of ICU stays and hospital stay, and overall cost were all significantly higher in patients who had deep vein thrombosis (*P* < 0.05, see Table 2). Furthermore, patients with postoperative coagulation function disorder, including increased FDP and D-dimer and decreased APTT, also had a significantly increased incidence of DVT (*P* < 0.05).

**Table 2:** Demographics, baseline values, and perioperative variables group by deep venous thrombosis.

	**Overall**	**DVT**	**Non-DVT**	***P*** **-value**
	***N*** = **456**	***n*** = **84**	***n*** = **372**	
Age, years, median (IQR)	56 (46–66)	66 (56–69)	54 (43–64)	<0.001
Age, > 60 years, no. (%)	173 (37.9)	55 (65.5)	118 (31.7)	<0.001
Gender, male, no. (%)	284 (62.3)	52 (61.9)	232 (62.4)	0.937
BMI, kg/m^2^, median (IQR)	24.2 (21.9–26.3)	24.2 (22.1–26.1)	24.2 (21.8–26.3)	0.936
**Blood type, no. (%)**				
ABO				0.114
A	113 (24.8)	29 (34.5)	84 (22.6)	
B	139 (30.5)	25 (29.8)	114 (30.6)	
AB	49 (10.7)	8 (9.5)	41 (11.0)	
O	155 (34.0)	22 (26.2)	133 (35.8)	
Rh+	456 (100.0)	84 (100.0)	372 (100.0)	1.000
**Medical history, no. (%)**				
Diabetes	50 (11.0)	14 (16.7)	36 (9.7)	0.065
Hypertension	100 (21.9)	22 (26.2)	78 (21.0)	0.296
Hyperlipidemia	4 (0.9)	0 (0.0)	4 (1.1)	1.000
Myocardial infarction	18 (3.9)	3 (3.6)	15 (4.0)	0.845
Cerebral infarction	6 (1.3)	2 (2.4)	4 (1.1)	0.343
**Medication history, no. (%)**				
Steroid	0 (0.0)	0 (0.0)	0 (0.0)	1.000
Tumor WHO classification, no. (%)				0.988
Gliomas, glioneuronal tumors, and neuronal tumors	429 (94.1)	79 (94.0)	350 (94.1)	
Anaplastic meningioma/ependymo-mas	6 (1.3)	1 (1.2)	5 (1.3)	
Lymphoma	9 (2.0)	2 (2.4)	7 (1.9)	
Metastatic tumors	11 (2.4)	2 (2.4)	9 (2.4)	
Melanoma	1 (0.2)	0 (0.0)	1 (0.3)	
Tumor WHO grade, no. (%)				0.325
iii	104 (22.8)	14 (16.7)	90 (24.2)	
iv	330 (72.4)	66 (78.6)	264 (71.0)	
UNCLEAR	22 (4.8)	4 (4.8)	18 (4.8)	
**Preoperative coagulation, median (IQR)**				
Preoperative FDP, mg/L	1.3 (0.8–1.9)	1.3 (0.9–2.2)	1.2 (0.8–1.8)	0.233
Preoperative D-D, mg/L	0.5 (0.4–0.8)	0.6 (0.4–0.9)	0.5 (0.3–0.7)	0.010
Preoperative abnormal D-D, yes	24 (5.3)	8 (9.5)	16 (4.3)	0.053
Preoperative PT, s	11.1 (10.7–11.6)	11.1 (10.7–11.7)	11.2 (10.8–11.6)	0.362
Preoperative INR	1.01 (0.96–1.05)	1.00 (0.96–1.05)	1.01 (0.96–1.05)	0.408
Preoperative APTT, s	29.7 (27.8–31.8)	29.1 (27.2–30.4)	29.8 (28.0–32.1)	0.001
Preoperative abnormal APTT, yes	25(5.5)	10 (11.9)	15 (4.0)	0.013
Preoperative Fbg, g/L	2.98 (2.58–3.40)	3.07 (2.80–3.41)	2.97 (2.54–3.40)	0.077
Leg paresis, no. (%)	0 (0.0)	0 (0.0)	0 (0.0)	1.000
Preoperative KPS, no. (%)				0.078
≥80	303 (66.4)	47 (56.0)	256 (68.8)	
50–70	145 (31.8)	35 (41.7)	110 (29.6)	
≤ 40	8 (1.8)	2 (2.4)	6 (1.6)	
**Intraoperative fluid**				
Total fluid, mL/kg/h, median (IQR)	8.13 (6.44–9.83)	8.05 (6.92–9.51)	8.18 (6.36–9.92)	0.784
Crystalloid, mL/kg/h, median (IQR)	6.39 (5.21–7.81)	6.53 (5.50–7.70)	6.35 (5.10–7.89)	0.454
Colloidal, mL/kg/h, median (IQR)	1.60 (1.13–2.21)	1.54 (1.19–2.14)	1.65 (1.09–2.23)	0.577
Blood loss, mL/kg/h, median (IQR)	0.75 (0.56–1.04)	0.81 (0.56–1.25)	0.74 (0.56–1.00)	0.103
Blood loss < 20% blood volume, no. (%)	442 (96.9)	78 (92.9)	364 (97.8)	0.017
Urine volume, mL/kg/h, median (IQR)	4.95 (3.66–6.72)	4.50 (3.32–5.93)	5.05 (3.69–6.94)	0.015
Red blood cell, no. (%)	26 (5.7)	17 (20.2)	9 (2.4)	<0.001
Fresh frozen plasma, no. (%)	27 (5.9)	18 (21.4)	9 (2.4)	<0.001
< 20% blood volume	17 (3.7)	12 (14.3)	5 (1.3)	<0.001
≥20% blood volume	10 (2.2)	6 (7.1)	4 (1.1)	0.040
Human fibrinogen, no. (%)	2 (0.4)	1 (1.2)	1 (0.3)	0.248
Length of operation, > 5 h, no. (%)	206 (45.2)	56 (66.7)	150 (40.3)	<0.001
Surgical position, no. (%)				0.272
Supine position	306 (67.1)	25 (59.5)	281 (67.9)	
Lateral position	150 (32.9)	17 (40.5)	133 (32.1)	
**Postoperative coagulation, median (IQR)**				
Postoperative FDP, mg/L	8.4 (4.4–19.9)	16.6 (6.7–35.8)	7.7 (4.0–16.5)	<0.001
Postoperative D-D, mg/L	4.9 (2.1–10.5)	9.4 (3.6–15.2)	4.4 (1.9–8.9)	<0.001
Postoperative PT, s	12.4 (11.7–13.0)	12.3 (11.7–13.2)	12.4 (11.7–13.0)	0.912
Postoperative INR	1.11 (1.06–1.18)	1.11 (1.06–1.18)	1.11 (1.06–1.18)	0.897
Postoperative APTT, s	26.7 (24.9–28.5)	25.6 (24.0–27.6)	27.0 (25.2–28.8)	<0.001
Postoperative Fbg, g/L	3.28 (2.76–3.81)	3.28 (2.36–3.91)	3.28 (2.80–3.78)	0.515
Hemocoagulase, no. (%)	110 (24.1)	23 (27.4)	87 (23.4)	0.440
**Postoperative complications, no. (%)**				
Pneumonia	23 (5)	12 (14.3)	11 (3.0)	<0.001
Hepatic dysfunction	68 (14.9)	16 (19.0)	52 (14.0)	0.239
Renal dysfunction	7 (1.5)	1 (1.2)	6 (1.6)	0.776
Central nervous system infection	57 (12.5)	13 (15.5)	44 (11.8)	0.361
Anemia	128 (28.1)	36 (42.9)	92 (24.7)	0.001
Electrolyte disorder	249 (54.6)	62 (73.8)	187 (50.3)	<0.001
**Health economics**				
Admission to ICU, no. (%)	309 (67.8)	73 (86.9)	236 (63.4)	<0.001
Duration in the ICU, hour, median (IQR)	19 (27–52)	60 (18–160)	18 (16–40)	<0.001
Hospital stay, day, median (IQR)	11 (8–15)	15 (11–21)	11 (8–14)	<0.001
Cost, ten thousand yuan, median (IQR)	7.9 (6.4–10.2)	10.2 (7.6–13.6)	7.6 (6.2–9.4)	<0.001

DVT, deep venous thrombosis; IQR, interquartile range; BMI, body mass index; WHO, World Health Organization; FDP, fibrinogen degradation product; D-D, D-dimer; PT, prothrombin time; INR, international standardized ratio; APTT, activated partial thrombin time; Fbg, fibrinogen; KPS, Karnofsky performance status; ICU, intensive care unit.

The demographics, baseline values, and perioperative variables are illustrated in Table 2. Patients older than 60 years or with preoperative abnormal APTT had a significantly higher incidence of DVT (*P* < 0.05). For patients with DVT, red blood cells, fresh frozen plasma transfusion or surgery duration longer than 5 h were significantly higher than those without (*P* < 0.05). The univariate and multivariate analyses for deep vein thrombosis are presented in Table 3. Finally, age more than 60 years (OR: 4.08, 95% CI: 2.48–6.73, *P* < 0.001), preoperative abnormal APTT (OR: 3.22, 95% CI: 1.39–7.44, *P* = 0.006), fresh frozen plasma used during the operation (OR: 11.0, 95% CI: 4.74–25.53, *P* < 0.001), duration of operation over 5 h (OR: 2.96, 95% CI: 1.80–4.87, *P* < 0.001), and admission to ICU (OR: 3.82, 95% CI: 1.96–7.46, *P* < 0.001) were independently associated with the incidence of deep vein thrombosis.

**Table 3:** Univariate and multivariate analyses for deep venous thrombosis.

	**Univariate analysis**			**Multivariate analysis**		
	**Crude OR**	**95% CI**	***P*** **-value**	**Adjusted OR**	**95% CI**	***P*** **-value**
Age > 60 years, yes	4.08	2.48–6.73	<0.001	3.98	2.30–6.88	<0.001
Preoperative D-D, mg/L	1.16	0.88–1.55	0.297			
Preoperative abnormal D-D, yes	2.34	0.97–5.67	0.059			
Preoperative APTT, s	0.858	0.79–0.93	<0.001			
Preoperative abnormal APTT, yes	3.22	1.39–7.44	0.006	2.81	1.06–7.42	0.037
Blood loss, < 20% blood volume	0.29	0.10–0.85	0.024			
Urine volume, mL/kg/h	0.85	0.76–0.95	0.004			
Red blood cell, yes	10.23	4.38–23.92	<0.001			
Fresh frozen plasma, yes	11.00	4.74–25.53	<0.001	6.85	2.73–17.18	<0.001
Length of operation, > 5 h	2.96	1.80–4.87	<0.001	2.36	1.34–4.16	0.003
Postoperative FDP, mg/L	1.03	1.01–1.04	<0.001			
Postoperative D-D, mg/L	1.04	1.02–1.07	0.002			
Postoperative APTT, s	0.863	0.79–0.95	0.001			
Admission to ICU, yes	3.82	1.96–7.46	<0.001	2.49	1.21–5.12	0.013
Duration in the ICU, h	1.00	1.00–1.00	0.715			

OR, odds ratio; CI, confidence interval; D-D, D-dimer; APTT, activated partial thrombin time; Fbg, fibrinogen; FDP, fibrinogen degradation product; ICU, intensive care unit.

**Figure 2:**
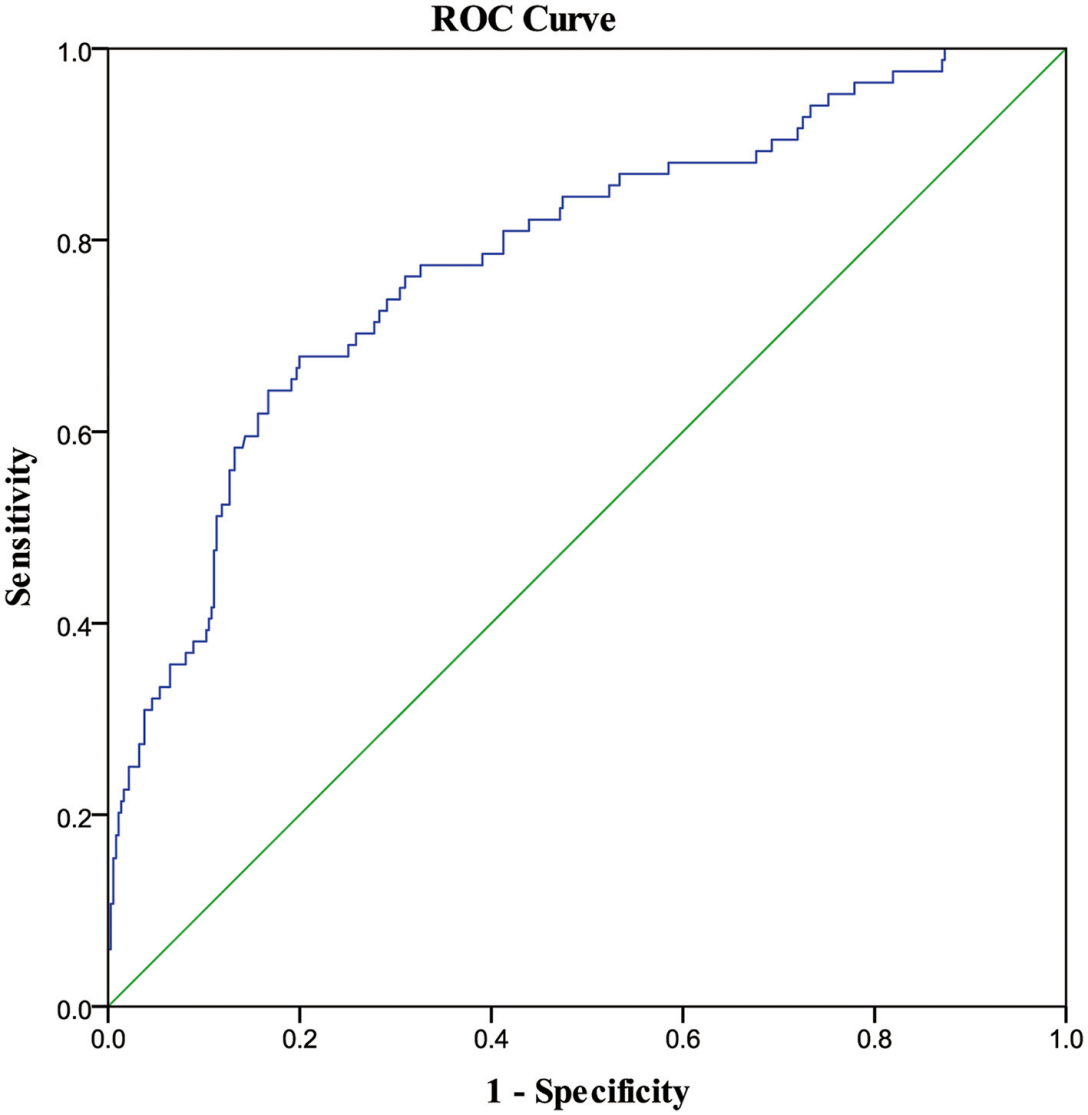
ROC curve of the risk factors associated with deep venous thrombosis. ROC, receiver operating characteristic curve; AUC, area under the curve. The blue curve represents the ROC curve of the multi-factor analysis model for postoperative risk factors associated with deep venous thrombosis. The AUC was 0.78 (95% CI: 0.73–0.84, *P* < 0.001).

The multivariate model indicated that intraoperative fresh frozen plasma transfusion (OR: 6.85, 95% CI: 2.73–17.18, *P* < 0.001) significantly increased the odds of the incidence of outcomes adjusted by the previously mentioned factors (Table 3). While assessing the model fit, the models demonstrated an average area under the curve of 0.78 (95% CI: 0.73–0.84, *P* < 0.001, see Figure 2).

## Discussion

For patients who underwent brain malignant tumors resection, age more than 60 years, fresh frozen plasma infusion, preoperative abnormal APTT, surgery duration longer than 5 h, or admission to ICU were significantly associated with the DVT.

In previous studies, the incidence of postoperative deep venous thromboembolism in patients who underwent brain tumor surgery has been reported to vary from 10.3 to 21.3% (3–7, 12). The DVT incidence in our study is 18.4%, which was consistent with a previous study that investigated 263 patients with high-grade gliomas (6). Recently, Shi et al. (7) measured the frequency of postoperative DVT in 373 high-grade glioma patients and reported an incidence of 16.1%. They only examined patients who exhibited symptoms or had increased D-dimer levels with ultrasound which might ignore asymptomatic patients with venous thrombosis. The incidence of postoperative DVT was found to be lower in patients with all types of brain tumors, and it ranged from 10.3 to 12.3% (3, 7, 12). One retrospective study reported that nine (0.54%) patients developed postoperative pulmonary embolism in 1,670 patients undergoing brain tumor surgery (7). Another retrospective study identified 107 (1.5%) postoperative pulmonary embolisms in 7,276 patients undergoing brain tumor surgery (8). However, no patients with postoperative pulmonary embolism were reported in our study. This difference may be due to the following reasons. We may fail to identify those patients with small pulmonary embolisms and mild symptoms which would be detected by computed tomography pulmonary angiogram. As computed tomography pulmonary angiogram was not routine in daily clinical practice, the incidence of pulmonary embolisms might be underestimated, which is also contributed by the relatively small sample size. Meanwhile, in previous reports, the incidence of perioperative cerebral ischemia in patients with glioma was reported to be 27.7–50% (9, 10), which included surgical cavity-related restricted diffusion. The incidence of cerebral ischemia in the current study was 9.2%, which was much lower than the previous study because we excluded the surgical cavity-related restricted diffusion.

For patients who underwent intracranial tumor resection, the mechanism of cerebral ischemia is more complicated. Vasospasms and blood vessel damage during surgery can cause local or extensive ischemia (13, 20). In patients with malignant brain tumors, in addition to the tumor compression of peripheral blood vessels or tumor blood steal (21, 22), cerebral ischemia may additionally be caused by tumor-induced inflammatory factors released into the periphery during tumor growth. However, the surgical procedure and inflammatory factors-related ischemia usually exist within or around the surgical field (23). To avoid interference with the surgical procedure, we defined postoperative cerebral ischemia as the one that is remote from the surgical cavity, excluding peritumoral ischemia. Therefore, surrounding vascular structures and the surgical approach were not selected for the model.

Preoperative lower extremity venous thrombosis is a high-risk factor for postoperative DVT. Ultrasound examination should be performed in patients with preoperative elevated D-dimer or a high risk of DVT (24). Among the 456 enrolled patients, 24 patients had preoperative abnormal D-dimer, of these four had a preoperative lower extremity venous ultrasound examination. DVT was confirmed in two patients as thrombosis progressed during postoperative reexamination of lower extremity venous ultrasound. Because preoperative venous ultrasonography was not performed routinely in our center, we were unable to detect the association between preoperative ultrasonography and postoperative DVT.

Due to the large size of the tumor, rich blood supply, and long operation time, it would be inevitable that the amount of intraoperative blood loss or extensive bleeding in the operative field would increase. Surgeons generally advocate the supplementation of fresh frozen plasma to reduce bleeding. Fresh frozen plasma contains all coagulation factors and is often used to supplement the deficiency of coagulation factors due to massive blood loss. However, over-aggressive infusion of fresh frozen plasma, such as using fresh frozen plasma when the bleeding volume is below the transfusion standard, may increase the risk of thrombosis. Our study indicated that for patients undergoing surgery for malignant craniocerebral tumors, intraoperative infusion of fresh frozen plasma significantly increased the incidence of thrombosis-related complications, when blood loss was less than 20% of blood volume. In our study, patients who received plasma infusion had significantly lower postoperative APTT (25.3 vs. 26.8 s, *P* = 0.014), suggesting the risk of postoperative hypercoagulability in transfused plasma. Our findings are consistent with the study of Kaewborisutsakul et al. (15), who examined 103 patients with extramedullary tumors undergoing surgical treatment and found that intraoperative use of fresh frozen plasma significantly increased the risk of postoperative thrombotic events (hazard ratio: 16.38, 95% CI: 1.47–182.23).

Hemocoagulase is often used to reduce the risk of hematoma after neurosurgery. It shortens the bleeding time with hemostatic function and does not affect the amount of prothrombin. However, hemocoagulase has been reported to have a tendency to increase the risk of deep venous thrombosis after abdominal surgery, although the difference was not statistically significant (23.3 vs. 10.0%, *P* > 0.05) (25). In our study, we did not find any impact of hemocoagulase use on postoperative thrombosis-related complications. In addition, human fibrinogen also affects coagulation function (26). In our cohort, only two patients were administered with human fibrinogen, so we could not draw any conclusion.

Patients with brain tumors may be in a hypercoagulable state. Brain parenchyma contains high expression levels of the tissue factor, a recognized procoagulant (27, 28). Inflammation and damage to the brain parenchyma may lead to the release of clotting agents in the blood of brain tumors. These clotting factors cause hypercoagulation throughout the body and increase the risk of developing blood clots. In our study, 25 (5.5%) patients already had APTT shortened preoperatively, representing endogenous coagulation abnormalities and the prethrombotic state. Our study indicates that patients with preoperative shortened APTT are more likely to develop deep vein thrombosis. At the same time, patients with deep vein thrombosis also had significantly shortened APTT after surgery, accompanied by significant increases in FDP and D-dimer. However, thrombosis-related complications may have started during postoperative coagulation examination, so the measurement of postoperative coagulation function could not be regarded as a risk factor or enter the model for the prediction of postoperative deep vein thrombosis.

Several studies have confirmed that the risk of postoperative deep vein thrombosis increases significantly with age (5, 11, 12). In our study, elderly patients (≥60 years old) were at a high risk of postoperative deep vein thrombosis. The duration of the operation also affects the incidence of postoperative deep vein thrombosis. A retrospective study found that surgical duration > 350 min was significantly associated with an increased risk of postoperative deep vein thrombosis (OR: 1.82, 95% CI: 1.27–2.60, *p* = 0.001) (7). In our cohort, we set the cutoff value at 5 h based on the median surgical duration of patients (4.8 h), and the results showed that surgery longer than 5 h significantly increased the odds of deep vein thrombosis, which is consistent with previous studies. Patients with brain malignant tumors often exhibit severe brain edema which is frequently treated with corticosteroids (29). However, some studies have shown that corticosteroids significantly increase the risk of postoperative venous thrombosis (30). Alternatively, mannitol is routinely administered to reduce cerebral edema in our center, and none of the patients we enrolled had long-term use of corticosteroids.

Our study has certain limitations. First, the enrolled patients were relatively healthier, with few preoperative complications. Second, as an observational study, it is impossible to determine the causal association. Third, this study only focused on coagulation parameters on the first postoperative day, more extended tracking of coagulation function is desirable for future studies. Finally, we failed to detect the risk of cerebral ischemia caused by thrombus detachment or paradoxical embolism through routine ultrasonography screening of the lower extremity veins or echocardiography. Similar to cerebral angiography, the preexisting brain arterial stenosis or occlusion that contributed to cerebral ischemia was not evaluated.

In summary, patients with malignant brain tumors have a high incidence of postoperative deep vein thrombosis and cerebral ischemia. Advanced age, preoperative abnormal APTT, surgery duration of more than 5 h, and admission to ICU were the independent risk factors of deep vein thrombosis. At the same time, intraoperative plasma infusion was associated with postoperative deep vein thrombosis and cerebral ischemia and therefore it should be cautiously administered. Perioperative monitoring should be intensified if fresh frozen plasma is indeed required. A further prospective study is needed to explore the influence of blood products on thrombosis-related complications after malignant brain tumor resection.

## Data availability statement

The original contributions presented in the study are included in the article/Supplementary material, further inquiries can be directed to the corresponding author.

## Ethics statement

The studies involving human participants were reviewed and approved by the Medical Ethics Committee of Beijing Tiantan Hospital, Capital Medical University (KY2021-173-02). Written informed consent for participation was not required for this study in accordance with the national legislation and the institutional requirements.

## Author contributions

YP designed the study. XZ, XL, MZ, and TM participated in data collection and manuscript preparation. XZ, ML, SL, LZ, and YP conducted the analysis. XZ, IK, and YP contributed to manuscript preparation. All authors reviewed and approved the manuscript.
