# Supplementary material for: Risk factors for postoperative thrombosis-related complications in patients undergoing malignant brain tumor resection: a retrospective cohort study
Source: Front Neurol. 2023 Apr 18;14:1108596. doi: 10.3389/fneur.2023.1108596 (PMC10151791; doi:10.3389/fneur.2023.1108596)
Supplement: Supplementary file 1 [file Table_1.docx]

Supplementary Material

Risk factors for postoperative thrombosis-related complications in patients undergoing malignant brain tumor resection: a retrospective cohort study

Xiaoyuan Liu^1†^, Xingyue Zhang^1†^, Tingting Ma^1^, Muhan Li^1^, Liyong Zhang^1^, Shu Li^1^, Min Zeng^1^, Ira S. Kass^2^, Yuming Peng^1^

*** Correspondence:** Yuming Peng: florapym766@163.com

# Supplementary Data

| Supplementary table 1. Definition of secondary outcomes. | |
| --- | --- |
| Outcomes | Definition |
| Myocardial infarction | Two of the following three criteria are met: typical chest pain, typical electrocardiogram, myocardial enzyme changes. |
| Arrhythmias | Diagnosis based on electrocardiogram. |
| Postoperative pneumonia | Various types of lung substantial inflammation caused by bacteria, fungi and other pathogens in hospitalized patients from 24 h after surgery to 2 weeks after surgery. |
| Abnormal liver function | Postoperative transaminase elevation or abnormal serum albumin level. |
| Abnormal renal function | Serum creatinine ≥ 0.3 mg/dL within 48 hours of surgery, or a ≥ 1.5 times increase from baseline within 7 days after surgery. |
| Central nervous system infection | Postoperative epidural abscess, subdural pus, meningitis, ventriculitis and brain abscess. Ventricle and lumbar cistern external drainage, shunt and implant-related meningitis or ventriculoencephalitis. |
| Anemia | Male hemoglobin <120g/L, Female hemoglobin <110g/L. |
| Electrolyte disturbances | Blood biochemical examination showed abnormal content of sodium ion, potassium ion and chloride ion |

| Supplementary table 2. Demographics, baseline values and perioperative variables group by cerebral ischemia. | | | | |
| --- | --- | --- | --- | --- |
|  | Overall | cerebral ischemia | Non-cerebral ischemia | P value |
|  | N=456 | n=42 | n=414 |  |
| Age, years, median (IQR) | 56 (46-66) | 60 (48-67) | 56 (45-66) | 0.200 |
| Age, > 60 years, no. (%) | 173 (37.9) | 21 (50.0) | 152 (36.7) | 0.091 |
| Gender, male, no. (%) | 284 (62.3) | 24 (57.1) | 260 (62.8) | 0.471 |
| BMI, kg/m^2^, median (IQR) | 24.2 (21.9-26.3) | 24.2 (21.9-26.2) | 24.2 (21.9-26.4) | 0.952 |
| Blood type, no. (%) |  |  |  |  |
| ABO |  |  |  | 0.105 |
| A | 113 (24.8) | 16 (38.1) | 97 (23.4) |  |
| B | 139 (30.5) | 14 (33.3) | 125 (30.2) |  |
| AB | 49 (10.7) | 3 (7.1) | 46 (11.1) |  |
| O | 155 (34.0) | 9 (21.4) | 146 (35.3) |  |
| Rh+ | 456 (100.0) | 42 (100.0) | 414 (100.0) | 1.000 |
| Coexisting medical condition, no. (%) |  |  |  |  |
| Diabetes | 50 (11.0) | 7 (16.7) | 43 (10.4) | 0.217 |
| Hypertension | 100 (21.9) | 8 (19.0) | 92 (22.2) | 0.636 |
| Hyperlipidemia | 4 (0.9) | 0 (0.0) | 4 (1.0) | 0.522 |
| Myocardial infarction | 18 (3.9) | 3 (7.1) | 15 (3.6) | 0.264 |
| Cerebral infarction | 6 (1.3) | 1 (2.4) | 5 (1.2) | 0.525 |
| Tumor WHO classification, no. (%) |  |  |  | 0.841 |
| Gliomas, glioneuronal tumors, and neuronal tumors | 429 (94.1) | 40 (95.2) | 389 (94.0) |  |
| Anaplastic meningioma/ependymo-mas | 6 (1.3) | 1 (2.4) | 5 (1.2) |  |
| Lymphoma | 9 (2.0) | 0 (0.0) | 9 (2.2) |  |
| Metastatic Tumors | 11 (2.4) | 1 (2.4) | 10 (2.4) |  |
| Melanoma | 1 (0.2) | 0 (0.0) | 1 (0.2) |  |
| Tumor WHO grade, no. (%) |  |  |  | 0.575 |
| III | 104 (22.8) | 8 (19.0) | 96 (23.2) |  |
| IV | 330 (72.4) | 33 (78.6) | 297 (71.7) |  |
| Unclear | 22 (4.8) | 1 (2.4) | 21 (5.1) |  |
| Preoperative coagulation, median (IQR) |  |  |  |  |
| Preoperative FDP, mg/L | 1.3 (0.8-1.9) | 1.4 (0.8-1.8) | 1.2 (0.9-1.9) | 0.967 |
| Preoperative D-D, mg/L | 0.5 (0.4-0.8) | 0.6 (0.4-0.7) | 0.5 (0.4-0.8) | 0.657 |
| Preoperative abnormal D-D, yes | 24 (5.3) | 2 (4.8) | 22 (5.3) | 0.879 |
| Preoperative PT, sec | 11.1 (10.7-11.6) | 11.1 (10.8-11.6) | 11.1 (10.7-11.6) | 0.765 |
| Preoperative INR | 1.01 (0.96-1.05) | 1.01 (0.96-1.05) | 1.01 (0.96-1.05) | 0.733 |
| Preoperative APTT, sec | 29.7 (27.8-31.8) | 29.3 (27.3-31.1) | 29.7 (27.8-31.8) | 0.281 |
| Preoperative abnormal APTT, yes | 25(5.5) | 2 (4.8) | 23 (5.6) | 0.830 |
| Preoperative Fbg, g/L | 2.98 (2.58-3.40) | 3.00 (2.77-3.28) | 2.98 (2.56-3.42) | 0.819 |
| Leg paresis, no. (%) | 0 (0.0) | 0 (0.0) | 0 (0.0) | 1.000 |
| Preoperative KPS, no. (%) |  |  |  | 0.465 |
| ≥80 | 303 (66.4) | 26 (61.9) | 277 (66.9) |  |
| 50-70 | 145(31.8) | 16 (38.1) | 129 (31.2) |  |
| ≤40 | 8 (1.8) | 0 (0.0) | 8 (1.9) |  |
| Intraoperative fluid |  |  |  |  |
| Total fluid, mL/kg/hour, median (IQR) | 8.13 (6.44-9.83) | 7.68 (6.91-9.01) | 8.16 (6.43-9.92) | 0.485 |
| Crystalloid, mL/kg/hour, median (IQR) | 6.39 (5.21 - 7.81) | 6.22 (5.30-7.18) | 6.41 (5.21-7.92) | 0.621 |
| Colloidal, mL/kg/hour, median (IQR) | 1.60 (1.13 - 2.21) | 1.73 (1.05-2.14) | 1.59 (1.13-2.23) | 0.734 |
| Blood loss, mL/kg/hour, median (IQR) | 0.75 (0.56 - 1.04) | 0.76 (0.61-1.04) | 0.75 (0.55-1.04) | 0.442 |
| Blood loss < 20% blood volume, no. (%) | 442 (96.9) | 39 (92.9) | 403 (97.3) | 0.108 |
| Red blood cell, no. (%) | 27 (5.9) | 5 (11.9) | 21 (5.1) | 0.069 |
| Fresh frozen plasma, no. (%) | 27 (5.9) | 7 (16.7) | 20 (4.8) | 0.002 |
| < 20% blood volume | 17 (3.7) | 5 (11.9) | 12 (2.9) |  |
| ≥20% blood volume | 10 (2.2) | 2 (5.8) | 8 (1.9) |  |
| Human fibrinogen, no. (%) | 2 (0.4) | 0 (0.0) | 2 (0.5) | 0.652 |
| Length of operation, > 5h, no. (%) | 206 (45.2) | 20 (47.6) | 186 (44.9) | 0.738 |
| Surgical position, no. (%) |  |  |  | 0.272 |
| Supine position | 306 (67.1) | 25 (59.5) | 281 (67.9) |  |
| Lateral position | 150 (32.9) | 17 (40.5) | 133 (32.1) |  |
| Postoperative coagulation, median (IQR) | | | | |
| Postoperative FDP, mg/L | 8.4 (4.4-19.9) | 8.9 (6.1-17.8) | 8.4 (4.2-20.0) | 0.525 |
| Postoperative D-D, mg/L | 4.9 (2.1-10.5) | 5.2 (3.3-10.3) | 4.9 (2.1-10.5) | 0.368 |
| Postoperative PT, sec | 12.4 (11.7-13.0) | 12.2 (11.7-13.0) | 12.4 (11.8-13.0) | 0.539 |
| Postoperative INR | 1.11 (1.06-1.18) | 1.10 (1.05-1.18) | 1.11 (1.06-1.18) | 0.532 |
| Postoperative APTT, sec | 26.7 (24.9-28.5) | 25.5 (24.0-27.7) | 26.8 (25.2-28.7) | 0.020 |
| Postoperative Fbg, g/L | 3.28 (2.76-3.81) | 3.30 (2.52-3.81) | 3.28 (2.77-3.81) | 0.607 |
| Hemocoagulase, no. (%) | 110 (24.1) | 8 (19.0) | 102 (24.6) | 0.420 |
| Postoperative complications, no. (%) |  |  |  |  |
| Pneumonia | 23 (5) | 3 (7.1) | 20 (4.8) | 0.514 |
| Hepatic dysfunction | 68 (14.9) | 5 (11.9) | 63 (15.2) | 0.566 |
| Renal dysfunction | 7 (1.5) | 0 (0.0) | 7 (1.7) | 0.396 |
| Central nervous system infection | 57 (12.5) | 5 (11.9) | 52 (12.6) | 0.903 |
| Anemia | 128 (28.1) | 17 (40.5) | 111 (26.8) | 0.060 |
| Electrolyte disorder | 249 (54.6) | 27 (64.3) | 222 (53.6) | 0.186 |
| Health economics |  |  |  |  |
| Admission to ICU, no. (%) | 309 (67.8) | 33 (78.6) | 276 (66.7) | 0.116 |
| Duration in the ICU, hour, median (IQR) | 19 (27-52) | 23 (18-76) | 19 (17-45) | 0.267 |
| Hospital stay, day, median (IQR) | 11 (8-15) | 13 (10-15) | 11 (8-15) | 0.063 |
| Cost, ten thousand yuan, median (IQR) | 7.9 (6.4-10.2) | 8.6 (6.8-11.1) | 7.8 (6.4-10.2) | 0.063 |
| IQR: interquartile range; BMI: Body Mass Index; WHO: World Health Organization; FDP: fibrinogen degradation product; D-D: D-dimer; PT: prothrombin time; INR: international standardized ratio; APTT: activated partial thrombin time; Fbg: fibrinogen; KPS: Karnofsky Performance Status; ICU: intensive care unit. | | | | |
